# Deep Learning Approach for Damage Classification Based on Acoustic Emission Data in Composite Materials

**DOI:** 10.3390/ma15124270

**Published:** 2022-06-16

**Authors:** Fuping Guo, Wei Li, Peng Jiang, Falin Chen, Yinghonglin Liu

**Affiliations:** 1 College of Mechanical Science and Engineering, Northeast Petroleum University, Daqing 163318, China; gfpmmc@163.com (F.G.); jpnepu@163.com (P.J.); honglin_7799@163.com (Y.L.); 2 Guangdong Provincial Key Laboratory of Petrochemical Equipment Fault Diagnosis, Guangdong University of Petrochemical Technology, Maoming 525000, China; chern.falin@outlook.com

**Keywords:** acoustic emission, composite material, deep learning, InceptionTime, damage classification

## Abstract

Damage detection and the classification of carbon fiber-reinforced composites using non-destructive testing (NDT) techniques are of great importance. This paper applies an acoustic emission (AE) technique to obtain AE data from three tensile damage tests determining fiber breakage, matrix cracking, and delamination. This article proposes a deep learning approach that combines a state-of-the-art deep learning technique for time series classification: the InceptionTime model with acoustic emission data for damage classification in composite materials. Raw AE time series and frequency-domain sequence data are used as the input for the InceptionTime network, and both obtain very high classification performances, achieving high accuracy scores of about 99%. The InceptionTime network produces better training, validation, and test accuracy with the raw AE time series data than it does with the frequency-domain sequence data. Simultaneously, the InceptionTime model network shows its potential in dealing with data imbalances.

## 1. Introduction

Carbon fiber-reinforced composites are widely used in various industrial sectors, such as in the aerospace, shipbuilding, automotive, and medical fields, due to their excellent properties of high temperature resistance, fatigue resistance, and low specific gravity [1,2,3]. However, variable operational and loading conditions may lead to damage, which can significantly jeopardize the safety of the structural system. Complicated failure mechanisms are distinct features of carbon fiber composites, and generally include intralaminar fiber breakage, matrix cracking, fiber/matrix interface debonding, and interlaminar delamination [4,5,6]. As composites experience deformation, a variety of damage modes interact with each other [7,8,9] and it is difficult to determine the different damage mode stages in composite materials. Consequently, it is necessary to perform damage onset detection, source localization, damage classification, and premature failure predictions on composite structures. There are many studies that have worked on these issues [10,11,12,13,14].

At present, acoustic emission (AE) technology has been attracting attention in structural health monitoring (SHM) [15,16,17,18,19]. It can dynamically, sensitively, and completely record the elastic waves generated by the irreversible changes in composite materials and convert them into electrical signals. After an in-depth analysis, it can reflect the damage present in materials and structures. A large number of AE features, including energy, duration, hits, amplitude, signal strength, peak frequency, rise time, and so on, can be extracted from the AE’s electrical signal [18,20,21,22,23]. All of these features can be used individually or together to [24,25] describe material and structural damage. When AE features are employed in certain combinations, damage mode identification can be achieved, and the AE characteristics of a specific damage mode can thus be discovered [17,24,25,26,27]. Other analysis methods are also used to seek more evidence of damage during AE data analysis, including cluster analysis [28], principal component analysis (PCA) [29], correlation coefficients, modal acoustic emissions [30,31], etc. The potential of AE technology in structural health monitoring has been proven.

With the development of big data, machine learning algorithms are used to relate the mechanical performance of composites to multiple AE features and to avoid the constrained interpretability of a single AE feature. Studying the waveform can help researchers understand damage characteristics better. In recent years, researchers have focused on developing automatic monitoring technologies using AE data [32,33]. Popularized by Hinton and others in the last decade, deep learning methods have achieved impressive success in image and speech recognition [32,34]. More researchers are applying deep learning techniques to deal with traditional fields with large amounts of data. Meanwhile, with the continuous update of AE instruments, which are equipped with multi-channel and broadband sensors and real-time full waveforms which contain AEs, big data are collected. Therefore, AE technology and deep learning are linked and have been adopted by many researchers [7,35,36,37,38,39,40,41]. The conversion of time series data into two-dimensional image data using short fast Fourier transform, wavelet transform, and the classification of acoustic emission data [42,43,44,45,46] using two-dimensional convolutional neural networks (CNNs) is a common method that has been used by many researchers.

Deep learning has been widely used in recent years for time series classification (TSC) data processing [47,48,49,50,51,52]. AE data are typically time series data, and applying deep learning to solve acoustic emission time series classification problems has not yet been reported upon or studied. This paper focuses on AE damage classification according to time series classification using the state-of-the-art InceptionTime model for the time series classification of datasets consisting of three types of single tensile failure AE data in carbon fiber-reinforced composites. Firstly, a series of laboratory experiments determining fiber breakage, matrix cracking, and delamination are carried out using the AE experimental system to obtain the AE datasets for the composite materials. Second, an InceptionTime model deep learning neural network with raw acoustic emission data, i.e., one-dimensional time series data as input, is constructed to perform damage classification in the selected composites. Third, the InceptionTime model deep learning network with frequency-domain sequences determined by fast Fourier transform (FFT) as input is constructed for training, validation, and testing to classify the damage in the composites. Finally, the classification performance of the IncepionTime model for the acoustic emission data is evaluated and discussed.

## 2. Experiment Procedures and Methods

### 2.1. Material Preparation and Test Procedure

Multiple failure modes such as fiber breakage, cracks in the matrix, fiber/matrix debonds, and delamination can occur in composite materials during loading. Oftentimes, two or more failure modes can develop together, leading to failure complexity or unpredictability. In most research work, fiber breakage, matrix cracking, and delamination have been studied as the main damage mechanisms [5,53,54,55]. The main objective of this paper is to apply a deep learning method for the damage classification of composite materials. Therefore, fiber bundle tensile tests, matrix tensile tests, and delamination single damage experiments were conducted in this study to obtain single failure data on fiber breakage, cracks in the matrix, and delamination in carbon fiber materials. The three experimental procedures were as follows.

**Figure 1:**
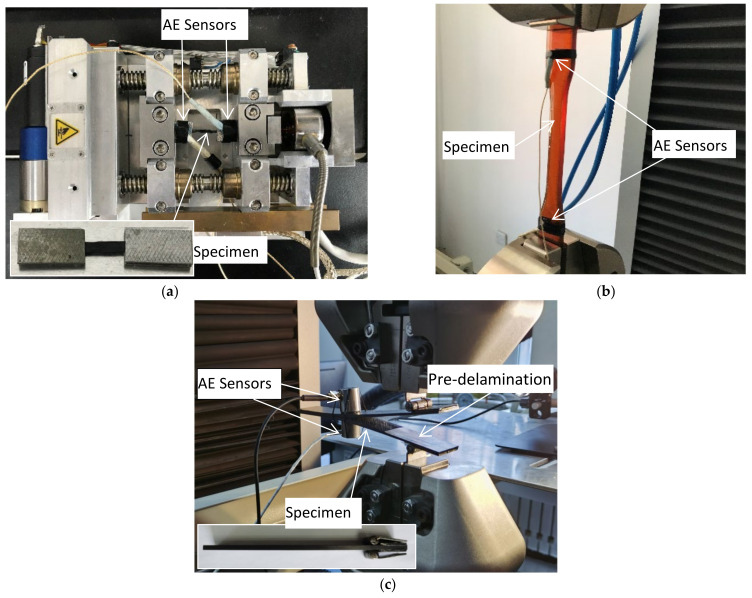
Experimental setup: (**a**) fiber bundle tensile test; (**b**) matrix tensile test; (**c**) delamination test.

The size of the carbon fiber tensile specimen was 45 mm × 3 mm (length × width) and was taken from a single-layer fiber bundle of T700SC-12000-50C with a 7 mm width. The detailed mechanical parameters of the material are shown in Table 1. In order to facilitate the acquisition of the acoustic emission signals during the stretching process and to observe the experimental process, the specimen was made using aluminum reinforcement sheets at both ends. Then, the SEM tester type in situ stretching machine was used to stretch the specimen and AE sensors were used for the reception of the propagating AE signals in the specimen in Figure 1a. Before the tensile test, noise sources in the surrounding environment were excluded, and the fiber bundles were continuously loaded at a speed of 5 mm/min until they fractured. The AE signal was obtained, and the damage pattern and experimental phenomena during the specimen failure were observed and recorded.

Because of its excellent overall performance, good processability, and low price, an epoxy resin matrix is currently the most commonly used resin matrix [57]. A tensile test was performed on the matrix according to the standard GB/T 2567-2008 [58]. An epoxy resin casting body with the dimensions 250 mm × 25 mm × 2.5 mm (length × width × height) and a dumbbell shape were selected for the matrix specimen. Then, the specimen was stretched using a Shimadzu AG-X electronic universal testing machine. The mechanical parameters of the epoxy resin matrix are shown in Table 2. Meanwhile, AE signals were continuously collected by the AE sensors during the test, as shown in Figure 1b. Pre-stretching was performed before formal stretching to reduce the friction signal between the specimen and the fixture. At the same time, the specimen was continuously loaded at a speed of 5 mm/min until the final fracture occurred. During the test, observation and recording of the damage pattern, loading curve and experimental phenomena during the specimen failure were carried out.

In order to control the bending stiffness of the thin plate to within a certain range, the combined effects of bending and torsion coupling, cantilever stiffness, and thermal residual stress were considered [5,59]. The delamination test specimen was selected from the carbon fiber composite laminate with the model number T700, and the laying mode of laminate was [0°]_24_ and had the dimensions 175 mm × 25 mm × 4 mm. The delamination damage test was performed according to mode I of the ASTM D5528-13 [60] standard. In this mode, delamination may include a large amount of fiber bridging [61]. According to the standard ASTM-D5528-13, a polytetrafluoroethylene (PTFE) film (55 mm × 25 mm × 40 μm) was inserted between layers 12 and 13 on the specimen to create initial delamination cracks. A pair of loading hinges was also glued to one end of the prefabricated crack to make a double cantilever beam (DCB) specimen. Subsequently, AE-based delamination damage tests were performed by applying tensile loads to the loading hinges, as shown in Figure 1c. Before the experiment started, the noise sources in the environment were first excluded, and then the tensile machine continuously loaded the DCB specimens at a speed of 1 mm/min until the specimen completely delaminated and failed. During this process, the acoustic emission signal was recorded, and the experimental phenomena were observed and recorded.

### 2.2. AE Data Acquisition Device

In the three experiments, the AE acquisition system consisted of an Express-8 acoustic emission detector, 2/4/6 preamplifier, WSα broadband sensor, and the specific configuration parameters shown in Table 3. Among them, two broadband AE sensors (α-series WSα SNAC88) were installed on the upper surface to receive the AE signals propagating in the specimen, and the acoustic emission signals were recorded in real time during the tests. The resonant frequency of the sensors was 125 kHz, and the optimum operating frequency ranges were 100–1000 kHz. Broadband acoustic emission sensors have a flat response in a certain frequency band and can reflect the original waveform intrinsically. A thin layer of the coupling agent filled the surface between the sensor and the specimen to increase the transmission rate of the acoustic emission waves and to achieve good acoustic coupling. The sensor was fixed in place with tape in the fiber bundle tensile test and matrix tensile test, while the sensor was fixed with highly elastic glue during the delamination test to effectively avoid the influence of noise.

### 2.3. Experimental Data and AE Datasets

**Figure 2:**
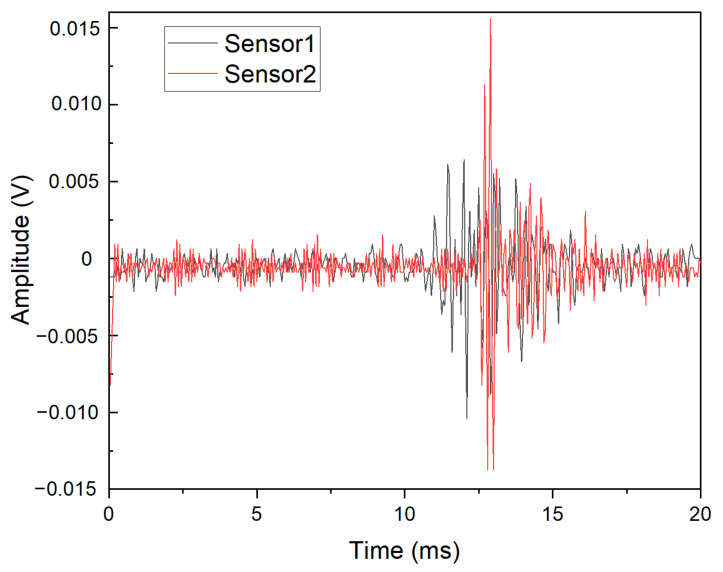
Raw AE signals (time domain) of fiber breakage.

According to the observations made during the experiment, it was found that the acoustic emission data were abundant for the fiber fracture and delamination experiments and relatively small for the matrix fracture experiment. Therefore, the AE data from 1 fiber tensile test, 1 delamination test and 5 matrix tensile tests were selected as the datasets for deep learning. Their numbers were 4500, 4500 and 216 AE raw time-domain data, respectively. The experimental AE signals (in time-domain) were obtained from the two sensors. The registered AE raw time-domain signals of fiber breakage for one experiment are presented in Figure 2.

AE data from three types of single-damage experiments: the fiber bundle tensile test, matrix tensile test, and delamination experiments, were constructed as AE datasets and labeled as C1, C2, and C3, respectively. Because the collection rate was 1MSPS, each AE hit generated a 1024-line dataset. The raw waveform multivariate time series were generated for the acoustic emission hits obtained by the acoustic emission acquisition system in the experiments. In total, 4500, 162, and 4500 sample data were obtained from the AE waveforms in the C1, C2, and C3 case datasets. It is worth pointing out that, as seen from Table 2, the epoxy resin matrix material was not resistant to stretching and cracking due to its brittleness and low toughness, resulting in relatively little AE data being obtained in the matrix tensile test. This means that the number of single experimental acoustic emission damage datasets obtained for the three types of composites in the experiment was imbalanced.

As seen in Section 1, two-dimensional CNNs have become popular for acoustic emission signal processing in recent years. The literature [45] has noted that the AE signals (time domain) in composite materials can be converted to time-frequency scalograms by performing continuous wavelet transform and that these scalogram images can be used as inputs to the CNN architecture while achieving high accuracies of 94.3–97.1%. Therefore, an interesting comparison was performed on the obtained datasets. We also attempted the proposed method and CNN architecture on our datasets, and the damage classification test accuracy was determined to be around 84.6%.

### 2.4. InceptionTime Model

In order to improve the accuracy of the damage classification tests on the obtained AE datasets, this thesis proposes the construction of a deep learning network using the InceptionTime model determined via time series classification to classify damage.

**Figure 3:**
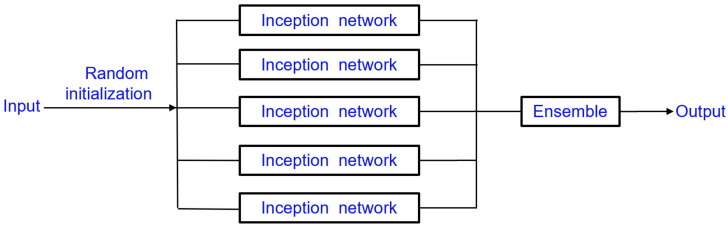
The ensemble of Inception networks.

**Figure 4:**
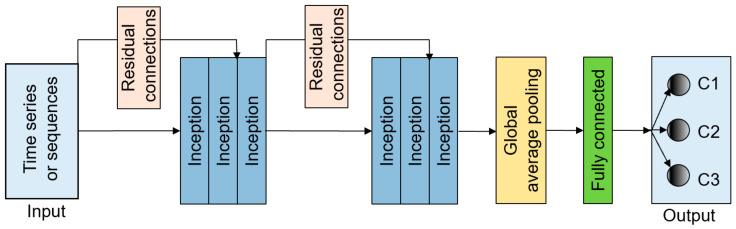
The adopted Inception network architecture for time series classification.

The InceptionTime model is an ensemble containing five different Inception networks that have been randomly initialized (Figure 3). One inception network (Figure 4) consists of six different inception modules that have been stacked and three inception modules are one residual block [62]. The input of each residual block is transferred via a shortcut linear connection to be added to the next block’s input, thus mitigating the vanishing gradient problem by allowing a direct flow of the gradient [63]. Next, a global average pooling (GAP) layer that averages the output multivariate time series over the whole time dimension is employed. Finally, a final traditional fully connected softmax layer with a number of neurons equal to the number of classes in the dataset is used.

**Figure 5:**
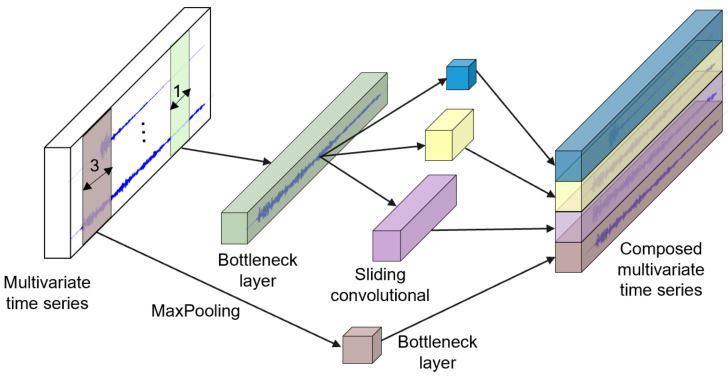
Inside InceptionTime module for TSC with a bottleneck layer of size m = 1.

The inside details of this operation in the Inception module are shown in Figure 5. The long green block in the middle of the figure, known as the bottleneck layer, is the first component, and thus the first major component of the Inception module. An operation using the sliding filters with a length of 1 and a stride equal to 1 is performed in this layer. This can help to transform the time series from a multivariate time series with M dimensions to another multivariate time series with m ≤ M dimensions. This reduces the dimensionality of the time series and minimizes the overfitting problem for small datasets. The second main component of the Inception module includes multiple synchronously sliding filters of different lengths on the same input time series. As shown in Figure 5, three different convolutions with the lengths 10, 20, and 40 are adopted as the multivariate time series input, which is also the output of the bottleneck layer [62]. Furthermore, another parallel MaxPooling operation is employed to make the model stable without it being affected by small perturbations. A bottleneck layer subsequently follows to reduce the dimensionality. The slidable MaxPooling output is calculated by taking the maximum value in that given time series window. Ultimately, the outputs of each independent parallel convolution and the MaxPooling output are concatenated and used as the output of the multivariate time series. The subsequent operations are duplicated for each initialization module of the InceptionTime model.

By stacking multiple Inception modules and then training the weights, i.e., the values of the filters, this deep learning network is able to extract latent features at multiple resolutions possible due to the use of multiple length filters. As such, there are three sets of thirty-two filters (with the lengths 10, 20, and 40) and MaxPooling added to the mix, thus making the total number of filters per layer equal to 32 × 4 = 128 = M, constituting the dimensionality of the output of the multivariate time series in the Inception module. The default value for the bottleneck size is set to m = 32, and the batch size is set to 8. A rectified linear unit (RELU) activation function is selected. This RELU activation function has been found to be advantageous in terms of speed and accuracy over the “sigmoid” and “tanh” function [64].

The following equation can explain the ensembling of predictions made by a network with different initializations [62] in the InceptionTime model:

(1) $${\hat{y}}_{i,c}=\frac{1}{n}\sum_{j=1}^{n}\sigma_{c}\left(x_{i},\theta_{j}\right)|\forall_{c}\in [1,C]$$

where ${\hat{y}}_{i,c}$ denotes the ensemble’s output probability of having the input time series $x_{i}$ belonging to class *c*, which is equal to the logistic output $\sigma_{c}$ averaged over n randomly initialized models. In [63], more details about ensembling neural networks for TSC can be found.

### 2.5. Methodology for InceptionTime Model-Based Damage Classification

**Figure 6:**
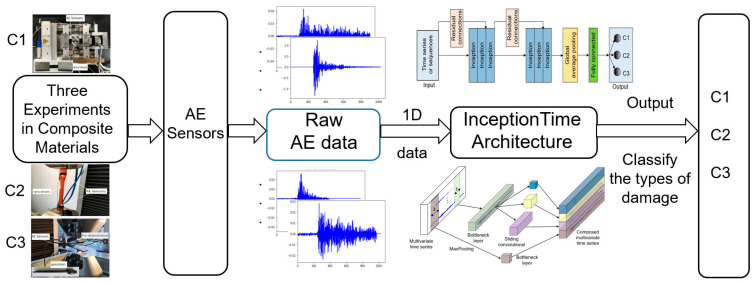
Schema of the InceptionTime deep learning model based on the raw AE time series data damage classification of composite materials.

The state-of-the-art deep learning InceptionTime model is applied to classify three types of damage, C1, C2, and C3, in composite materials. In contrast to the traditional method of extracting features from AE data, the use of raw acoustic emission data, i.e., time series data, is employed as the input to the neural network. Figure 6 shows a schematic of the InceptionTime-based damage classification approach for the current problem.

The proposed InceptionTime model was trained, validated, and tested in the InceptionTime architecture using the raw AE data received from the AE sensors in the acoustic emission experiments, i.e., the raw AE time series one-dimensional data, as input. The model outputs were three classifications of composite damage (Figure 6).

To achieve stable network performance, 100 training, validation, and testing epochs were applied. During the process, for the labeled datasets, a total of 9162 samples were divided into the training set, validation set, and test set in the ratio of 6:2:2. This means that all of the AE time series data that had been artificially labeled to the three damage classes (C1, C2, C3) were divided into ten parts, six of which were used for training, two of which were used for validation, and two of which were used for testing.

**Figure 7:**
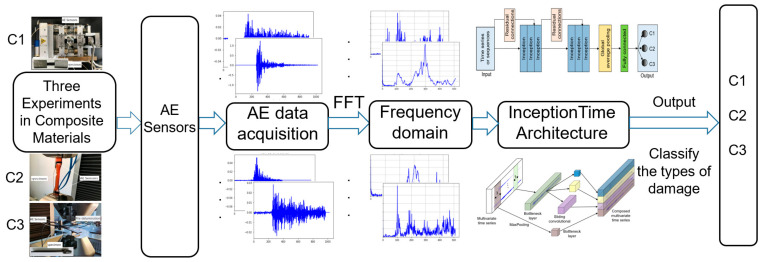
Schema of InceptionTime deep learning model based on frequency-domain sequences for the damage classification of composite materials.

**Figure 8:**
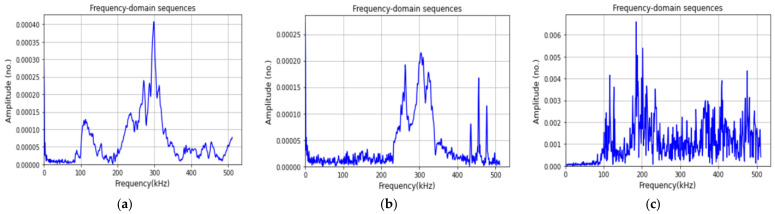
Several typical frequency-domain sequences determined by FFT: (**a**) fiber breakage, (**b**) matrix cracking, (**c**) delamination.

Furthermore, other sequence data from experimental datasets were considered to validate the classification performance of the InceptionTime model. Therefore, the registered time-domain AE signals from the experiments were converted to the frequency domain by fast Fourier transform (FFT), and these frequency-domain sequences were used as inputs to the proposed InceptionTime network for training, validation, and testing to classify the damage in the composites, as shown in Figure 7. Figure 8 represents some typical frequency-domain sequences obtained from a raw time-series waveform signal by means of FFT.

## 3. Results and Discussion

### 3.1. Train

We conducted an experiment on a computer with Intel Xeon Bronze 3106 @ 1.7 Ghz, 64-GB RAM, 2 NVIDIA TITAN Xp (1582 MHz, 3840 CUDA cores, 12 GB). The code was written in Python 3.6.15 (Amsterdam, The Netherlands), Tsai 0.3.0, and TensorFlow 2.8 with CUDA 11.2. In deep learning, the hyperparameter is the parameter that is set before the learning process begins. These parameters are tunable and can directly affect how well a model is trained [65,66]. An empirical hyperparameter is a manually configurable setting that needs to be assigned the “correct” value based on existing or current experience, i.e., a value is artificially set for it. Some empirical hyperparameters in this study were set as follows: batch size = 8; learning rate = 0.001; epochs = 100.

#### 3.1.1. Raw AE Time Series Data

Initial damage mode identification efforts only used one or a few features of the waveform to describe the emitting damage mode [67,68]. In recent years, based on our prior knowledge of AE signals, deep learning methods have been developed by combining feature evaluation algorithms [17,23,24]. While these low-dimensional representations are useful for indicating broader trends in damage mode shifts, they obstruct the high-fidelity characterization of signals [69]. Raw AE time-domain waveform data can be used to classify damage patterns [70,71].

**Figure 9:**
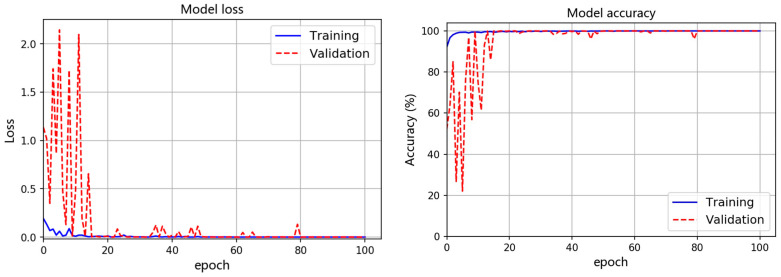
A typical training–validation curve showing the comparison of the accuracy and loss during the training and validation of InceptionTime network using raw AE time series data.

Using the raw acoustic emission time-domain data as the input of the InceptionTime model, model loss can be used to optimize the parameters to achieve gradient descent [72,73], which is the loss value calculated from a predefined loss function. The model accuracy is used to measure the metrics of the network and represents the evaluation results of the model on the dataset based on a given label. One of the training–validation results is presented in Figure 9 and shows the average course of validation accuracy and loss using raw AE time series data. The InceptionTime network was trained for 100 epochs, and no overfitting occurred. The validation accuracy reached convergence in about 38 epochs, which is evident from the decreasing loss in Figure 9.

The InceptionTime network for AE one-dimensional CNNs continuously improves its confidence by achieving accurate predictions. In the early epochs, because of the limited data used in a single batch, some fluctuations or oscillations can be observed in the validation accuracy and loss curves. Such training and validation were repeated, and the results of a total of 10 rounds show that the average accuracy of 10 times training/validation = {(97.6 + 100 + 99.73 + 99.89 + 100 + 99.95 + 100 + 99.95 + 100 + 100)/10} = 99.71%.

#### 3.1.2. Frequency-Domain Sequence Data

The broadband acoustic emission sensors enabled the full frequency spectrum of the signal to be recorded and the waveform to be digitally stored. It is not standard practice to perform a frequency spectrum analysis of the acoustic emission signals collected by broadband AE sensors [44,45].

**Figure 10:**
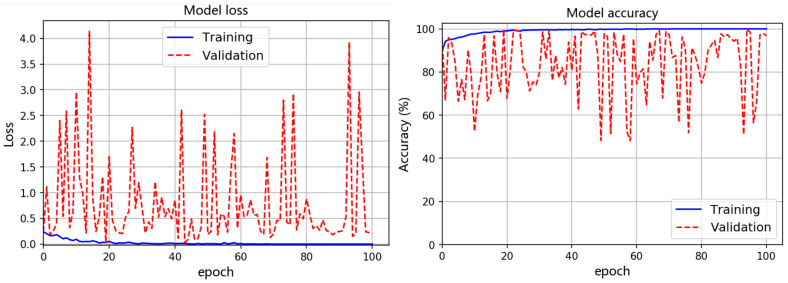
A typical training–validation result of the InceptionTime network using frequency-domain sequence data.

Figure 10 shows a typical training–validation curve showing the comparison of the accuracy and loss during the training and validation of the InceptionTime network with the frequency-domain sequence data. The validation accuracy mainly fluctuates between 60% and 99%, which is obvious from the model’s accuracy curve. Such training and validation were repeated, and the results of a total of 10 rounds show that the average accuracy over 10 training/validation times training/validation accuracy = {(96.73 + 97.87 + 99.38 + 98.36 + 99.32 + 99.25 + 98.77 + 98.98 + 99.52 + 96.86)/10} = 98.5%, which is lower than the result (i.e., 99.71%) obtained for the InceptionTime model when raw time was used as the input. Furthermore, a comparison between the training validation results corresponding to the raw time series and frequency-domain sequences showed that the InceptionTime model demonstrated consistently higher performance for the raw AE time series data.

### 3.2. Evaluation Metrics

This study used evaluation metrics that are appropriate for a classification study. The confusion matrix is a performance metric for machine learning classification problems [74] and is used here to evaluate the effectiveness of the InceptionTime model for classification problems.

Accuracy evaluates the overall quality of the damage classification. Since the number of samples was imbalanced, precision was used to evaluate the quality of the true positives that obtained positive predictions, recall was used to evaluate the quality of the positive predictions, and specificity was used to evaluate the quality of the negative predictions. The F1 score was computed to be between 0 and 1, which is the harmonic mean of precision and recall. They were calculated as follows:

(2) $$Accuracy=\frac{TP+TN}{TP+TN+FP+FN}$$

(3) $$\mathrm{Pr}ecision=\frac{TP}{TP+FP}$$

(4) Recall=TPTP+FN

(5) $$Specificity=\frac{TN}{TN+FP}$$

(6) F1−score=2×(Precision×Recall)Precision+Recall

where *TP* is the true positive, *FP* is the false positive, *TN* is the true negative, and *FN* is the false negative.

**Figure 11:**
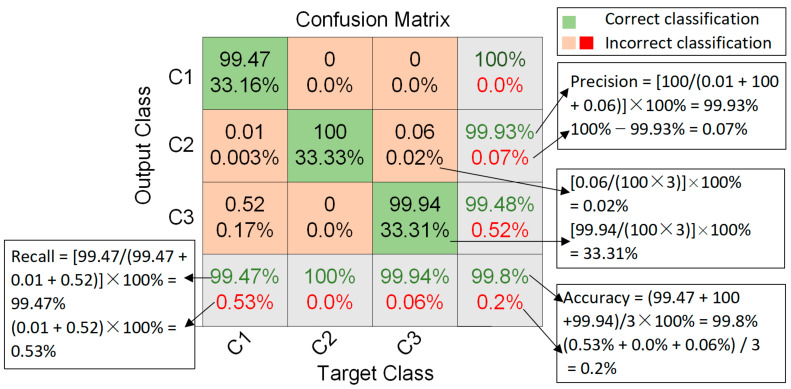
The confusion matrix denotes the test performance of the InceptionTime network using raw AE time series.

The confusion matrix in Figure 11 shows the test performance of the InceptionTime network using raw AE time series. The confusion matrix lists the calculation of the predicted overall test accuracy as the test accuracy = (99.47 + 100 + 99.94)/3 × 100% = 99.8%. Figure 11 lists the detailed calculation process to determine precision using C2 as an example, that is, precision = [100/(0.01 + 100 + 0.06)] × 100% = 99.93%. C1 and C3 can be calculated according to the precision analogy, with C1 and C3 achieving 100% and 99.48% accuracy. The detailed calculation process to determine the recall is listed in Figure 11 and uses C1 as an example, i.e., Recall = [99.47/(99.47 + 0.01 + 0.52)] × 100% = 99.47%; C2 and C3 can be calculated according to the recall analogy, with C2 and C3 achieving 100% and 99.94% recall.

Simultaneously, according to Equations (5) and (6), the specificity of C1, C2, and C3 can be calculated as 100%, 99.97%, and 99.5%, respectively, and the F1 scores of C1, C2, and C3 were determined to be 99.74%, 99.18%, 99.71%, respectively. The accuracy, precision, recall, specificity, and F1 score values were all relatively high—above 99%—which means that the InceptionTime model can achieve excellent classification results for the three types of acoustic emission raw sequence data for the single fiber fracture, matrix cracking, and delamination of composite materials.

**Figure 12:**
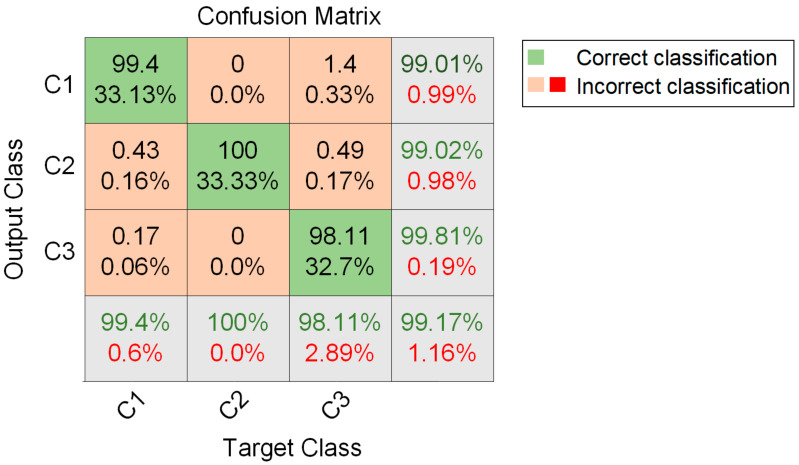
The confusion matrix denotes the test performance of the InceptionTime network using frequency-domain sequence data.

The confusion matrix in Figure 12 shows the test performance of the InceptionTime network using frequency-domain sequences. The confusion matrix lists the overall test accuracy of the InceptionTime model predictions based on the frequency-domain sequences as 99.17%. The precision values of C1, C2, and C3 were 99.01%, 99.02%, and 99.81%, respectively. The recall of values C1, C2, and C3 were 99.4%, 100%, and 98.11, respectively. According to the calculation of Equations (5) and (6), the specificity of C1, C2, and C3 can be calculated as 98.63%, 99.54%, and 99.84%, respectively, and the F1 scores of C1, C2, C3 were 99.2%, 99.51%, and 98.95%, respectively.

The accuracy, precision, recall, specificity, and F1 scores were above 98%, indicating that the InceptionTime model also had relatively good classification ability when using the acoustic emission frequency-domain sequence data for the fiber breakage, matrix cracking, and delamination of composite materials.

### 3.3. Comparative Analysis

In Section 2.3, the obtained AE datasets were used with the method and CNN architecture proposed in [45], and the damage classification test accuracy was around 84.6%, while in Section 3.2, the test accuracy of the method proposed in this paper was around 99%. This indicates that the deep learning method proposed in this paper greatly improves the damage classification accuracy for AE data in composite materials.

**Figure 13:**
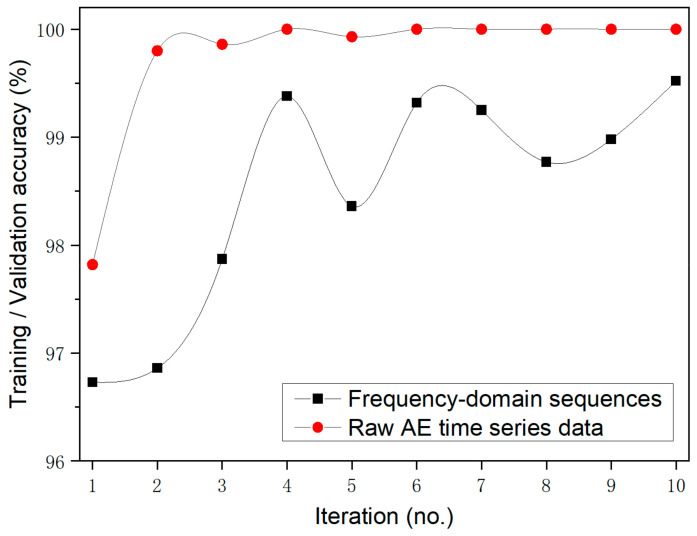
Comparing average test accuracy over 10 tests using the Inception network for raw AE time series data and frequency-domain sequences.

Furthermore, a comparison of average test accuracy over 10 tests using the Inception network for raw AE time series data and frequency-domain sequence data is presented in Figure 13. The figure clearly shows that the test accuracy of the InceptionTime model based on the raw AE time series data was close to 98% for one point and above 99.5% for the other nine points. The test accuracy of the InceptionTime model based on frequency-domain sequences was below 99.5% in all cases. The performance of the InceptionTime model based on the raw AE time series data was consistently higher than that of the InceptionTime model based on frequency-domain sequences.

**Figure 14:**
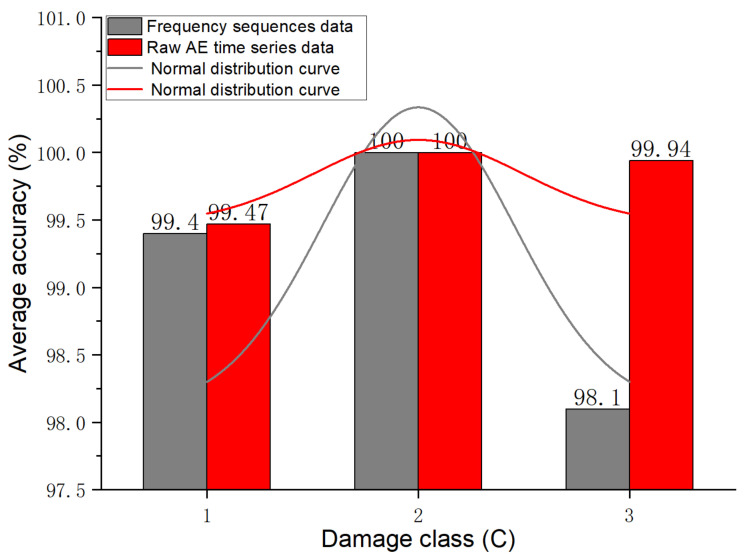
Comparison of the average test accuracy based on the raw AE time series data and frequency-domain sequence data for C1, C2, and C3.

Furthermore, a comparison of the average test accuracy over 10 test results corresponding to the raw AE time series data and test accuracy results corresponding to the frequency-domain sequence data is presented in Figure 14, and indicates a significant increase in the raw AE time series data results over the frequency-domain sequence data.

It is important to reiterate that in Section 2.3, 162 AE waveform sample data were obtained for the matrix tensile test, i.e., the C2 dataset, and any data augmentations were not performed on the C2 dataset. It was unbalanced with the number of samples in datasets C1 and C3. The unbalanced C1, C2, and C3 datasets were fed into the designed InceptionTime network for classification. Figure 14 is a comparison of the average test accuracy based on the raw AE time series data and frequency-domain sequence data for C1, C2, and C3. The figure shows that for category C1, fiber breakage, the average test accuracies of the raw frequency-domain sequence data and AE time series data were 99.4 and 99.47, respectively. For category C2, matrix cracking, the average test accuracies of the raw AE time series data and frequency-domain sequence data were both 100%. For category C3, i.e., delamination, the average test accuracies of the raw AE time series data and frequency-domain sequence data were 98.11 and 99.94, respectively. Figure 14 also shows the exciting fact that although the C2 dataset had fewer samples and did not have any expansion, the InceptionTime model nevertheless achieved 100% accuracy for the C2 classification effect.

## 4. Conclusions

In this paper, a deep learning network based on the state-of-the-art InceptionTime model was established for damage classification in composite materials. The datasets used in this study consisted of three types of single tensile failure AE data in a carbon fiber-reinforced composite. There were 4500, 162, and 4500 AE sample data in the fiber breakage (i.e., C1), matrix cracking (i.e., C2), and delamination (i.e., C3) case datasets, respectively. AE raw time series and frequency-domain sequence data were employed as inputs for the deep learning approach for automatic damage classification in composite materials. The following conclusions can be drawn.

The test confusion matrix of the InceptionTime network revealed that the proposed deep learning approach for AE damage classification can effectively distinguish the three tensile damage type classes of fiber breakage, matrix cracking, and delamination in composite materials, achieving a high test accuracy of about 99%.

The average training/validation accuracy of the raw AE time series data as input to the InceptionTime network was 99.71%, and the test accuracy was 99.8%. The average training/validation accuracy was 98.5%, and the test accuracy was 99.17% when the frequency-domain sequence data were used as the input for the InceptionTime network. Both can be used to obtain a very high classification performance. However, the proposed InceptionTime network produced better training, validation, and test accuracy when the raw AE time series data were used rather than the frequency-domain sequence data. Using raw AE time series data as the input for a deep learning network demonstrated excellent classification performance and great potential.

The matrix cracking acoustic emission dataset had only 162 data samples, while the fiber breakage and delamination datasets both had 4500 acoustic emission data samples. However, the InceptionTime model achieved 100% accuracy when classifying the matrix cracking data, even though there were only a few data samples available. The InceptionTime model network can solve data imbalance problems in AE datasets, providing a reference method for imbalanced data classification problems caused by only a small amount of sample data being available in practical engineering applications.

## Figures and Tables

**Table 1:** Mechanical parameters of the T700SC-12000-50C carbon fiber bundle [56].

Parameters	Average Value
Tensile strength/GPa	4.9
Tensile modulus/GPa	230
Elongation %	2.10
Density/kg/m^3^	1800
Poisson’s ratio	0.307
Diameter/μm	7

**Table 2:** Mechanical parameters of the epoxy resin matrix [58].

Parameters	Average Value
Tensile strength/MPa	75
Tensile modulus/GPa	3
Elongation %	0.2
Density/(kg/m^3^)	980
Poisson’s ratio	0.38

**Table 3:** AE parameters.

Parameter	Setting Value
Threshold/dB	35
Sampling rate/MSPS	1
Pre-trigger time/μs	256
Peak definition time (PDT)/μs	100
Hit definition time (HDT)/μs	200
Hit locking time (HLT)/μs	400

## Data Availability

Data can be provided upon request from the corresponding author.
